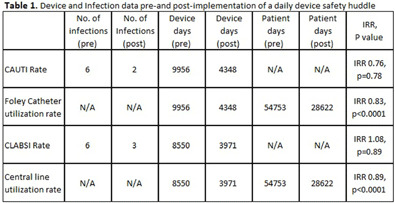# Impact of a Daily Device Safety Huddle on Device Utilization and Infections

**DOI:** 10.1017/ash.2025.399

**Published:** 2025-09-24

**Authors:** Jennifer Finn, Tiana Walters-Mitchell, Heather Rand, Iman Miles, Jacqueline Wooters, Anupama Neelakanta

**Affiliations:** 1 Atrium Health Pineville; 2 Atrium Health Pineville; 3 Atrium Health; 4 Atrium Pineville Health; 5 Atrium Healthcare; 6 Atrium Health

## Abstract

**Background:** Device-associated infections, such as catheter-associated urinary tract infections (CAUTI) and central line-associated bloodstream infections (CLABSI), increase patient mortality, morbidity, and length of stay. These infections are best prevented through appropriate use and maintenance of the devices. There is often a lack of accountability regarding the appropriateness of central lines and urinary catheters. Our team’s goal was to develop an approach that validates device necessity each day to reduce device utilization and ultimately decrease CAUTIs and CLABSIs. **Methods:** A multidisciplinary team, including infection prevention (IP), facility leaders, unit nursing leaders, performance improvement coordinators, and providers, implemented a hospital-wide (excluding the neonatal intensive care and maternity units) daily Device Safety Huddle (DSH), in a 360-bed hospital in September 2024. IP facilitates the meeting, and unit leaders or their delegates are expected to report daily on the number of central lines and urinary catheters on their unit. Leaders also report any concerns related to site or type, actual necessity, plans for removal, and barriers to removal. IP spot checks various charts to ensure that device necessity correlates with unit reporting. The team compared device utilization rates (DUR) and infection rates for CAUTI and CLABSI pre (January - August 2024) and post-intervention (September - December 2024). Statistical analysis was applied to assess the differences between both groups. **Results:** DUR for urinary catheters reduced from 18.1 per 100 patient days pre-intervention to 15.1 (IRR 0.83, p < 0 .0001) post-intervention, with similar reductions calculated for central lines from 15.6 per 100 patient days to 13.8 (IRR 0.89, p < 0 .0001). Infection rates remained stable for CAUTI (0.57 vs 0.43/1000 catheter days, IRR 0.73, p=0.77) and CLABSI (0.69 vs 0.74/1000 central line days, IRR 1.07, p=0.89) post-intervention (Table 1). **Conclusions:** Implementing a daily DSH helped improve accountability related to device necessity and decreased device utilization. The infection rate changes are not statistically significant at this time and will continue to be evaluated for long-term impact. The inclusion of administrative and director-level leadership is essential for accountability and the success of the intervention.

**Figure:**